# Targeting SARS-CoV-2 using polycomb inhibitors as antiviral agents

**DOI:** 10.2217/epi-2020-0154

**Published:** 2020-06-04

**Authors:** Sameer Ayaz, Francesco Crea

**Affiliations:** 1 School of Life Health & Chemical Sciences, The Open University, Walton Hall, Milton Keynes, UK

**Keywords:** COVID-19, epigenetics, EZH2, polycomb, SARS-Cov-2

SARS-CoV-2, a betacoronavirus is the infectious viral agent causing the current pandemic of COVID-19. The disease causes acute respiratory distress syndrome, an inflammatory response which is the major cause of mortality of this disease. The current pandemic has been projected to cause hundreds of thousands of deaths worldwide [1]. Currently, even with social distancing control strategies in place, the predicted death toll is still within the hundreds of thousands [2]. It is therefore of paramount importance to explore any possible diagnostic and therapeutic option that may reduce the burden of disease. The definitive solution to this global crisis will likely come from the development of an effective vaccine which is likely to take longer than one year [3]. In the meantime, it is important to evaluate a variety of therapeutic targets which can improve the prognosis of patients with severe COVID-19 infections, particularly to reduce the need for intensive care units, which have been quickly overwhelmed in many countries.

Several pharmacological approaches are being evaluated as therapeutics to reduce disease burden until effective vaccines are developed. The primary focus has been to repurpose approved therapeutics to target COVID-19 by using anti-inflammatories and antivirals. The aim of the first approach is to reduce the overactive inflammatory response to SARS-CoV-2 which has been suggested as a mechanism of increased disease severity [4], while the latter approach directly inhibits viral replication. Examples of such therapies currently in clinical trials are Salirumab (clinical trial code: NCT04315298; Regeneron Pharmaceuticals, NY, USA; Sanofi, Paris, France), a monoclonal antibody to block the IL-6 pathway to reduce the inflammatory response to infection and Remdesevir™ (clinical trial code: NCT04292730; Gilead Sciences, CA, USA), a terminating nucleotide analogue which inhibits viral replication and has recently shown *in vitro* activity against SARS-CoV-2 [5].

In this context, we would like to discuss an interesting link between druggable epigenetic modifications and the innate immune response mediated by Type I interferons (IFNs). The Type I IFN pathway is a key antiviral response induced early on in infection via host sensing of pathogen-associated molecular patterns which are typically aberrant nucleic acid structures not normally present in the host. The pathway ultimately leads to the transcriptional activation of interferon stimulated genes which are potent antiviral effectors.

A recent study showed that pathogenic influenza A viruses and other betacoronaviruses (MERS-CoV and SARS-CoV) inhibit the activation of IFN-dependent genes in infected cells [6] which in turn increases the pathogenicity of these viruses. Mechanistically, these viruses activate several epigenetic effectors, including the polycomb repressive complex 2 (PRC2), which mediates H3K27me3 at the promoter of certain IFN-stimulated genes repressing transcription. Interestingly, PRC2 has been shown to mediate a similar effect in cancer cells, where this complex represses the expression of MHC-I genes in an interferon-dependent way [7]. It is not known whether SARS-CoV-2 employs similar mechanisms to repress the IFN pathway in infected cells, but since MERS-CoV and SARS-CoV viruses are closely related, this hypothesis is plausible and should be further investigated.

Pharmacologic inhibitors of PRC2 are currently in advanced clinical trials for several malignancies [8] and could be therefore easily repurposed in the fight against COVID-19. Experiments targeting enhancer of zeste-1 homologue 2 which forms part of the PRC2 complex have shown that this enzyme plays a role in modulating viral replication by inhibiting replication of viruses such as Influenza A and Herpes Simplex 1 Virus using knockdown experiments [9] and small molecule inhibitors [10]. PRC2 inhibitors have also been shown to improve the activation of natural killer cells [11], which constitute one of the first lines of defence against viral infection [12]. In addition, some studies suggest that PRC2 activity is essential for the correct maturation of B and T lymphocytes [13].

Therefore, inhibitors targeting the PRC2 complex could be used to counteract viral repression of interferon stimulated genes and enhance the adaptive immune response in the case of SARS-CoV-2 infection. When testing the antiviral properties of PRC2 inhibitors, it will be essential to evaluate the optimal timing and duration of treatment and its use in combination with other therapies. In addition, most of the data on PRC2 inhibitors have been collected from cancer models so it will be important to study the role of these and other epigenetic drugs in the specific context of SARS-CoV-2 infection in relevant models. However, we think that the current situation requires every scientist to contribute new and possibly unconventional ideas, in the hope that some will soon produce effective treatments.
